# Pasireotide Therapy of Multiple Endocrine Neoplasia Type 1–Associated Neuroendocrine Tumors in Female Mice Deleted for an Men1 Allele Improves Survival and Reduces Tumor Progression

**DOI:** 10.1210/en.2015-1965

**Published:** 2016-03-18

**Authors:** Gerard V. Walls, Mark Stevenson, Benjamin S. Soukup, Kate E. Lines, Ashley B. Grossman, Herbert A. Schmid, Rajesh V. Thakker

**Affiliations:** Academic Endocrine Unit (G.V.W., M.S., B.S.S., K.E.L., R.V.T.), Radcliffe Department of Medicine, Oxford Centre for Diabetes, Endocrinology and Metabolism (OCDEM), University of Oxford, Churchill Hospital, Oxford, OX3 7LJ, United Kingdom; Nuffield Department of Surgical Sciences (G.V.W., B.S.S.), University of Oxford, John Radcliffe Hospital, Oxford, OX3 9DU, United Kingdom; Department of Endocrinology (A.B.G.), OCDEM, Churchill Hospital, Oxford, OX3 7LJ, United Kingdom; and Novartis Pharma AG (H.A.S.), Novartis Institutes for Biomedical Research, Oncology, CH-4057 Basel, Switzerland

## Abstract

Pasireotide, a somatostatin analog, is reported to have anti-proliferative effects in neuroendocrine tumors (NETs). We therefore assessed the efficacy of pasireotide for treating pancreatic and pituitary NETs that develop in a mouse model of multiple endocrine neoplasia type 1 (MEN1). *Men1*^+/−^ mice were treated from age 12 mo with 40 mg/kg pasireotide long-acting release formulation, or PBS, intramuscularly monthly for 9 mo. The *Men1*^+/−^ mice had magnetic resonance imaging at 12 and 21 mo, and from 20 mo oral 5-bromo-2-deoxyuridine for 1 mo, to assess tumor development and proliferation, respectively. NETs were collected at age 21 mo, and proliferation and apoptosis assessed by immunohistochemistry and TUNEL assays, respectively. Pasireotide-treated *Men1*^+/−^ mice had increased survival (pasireotide, 80.9% vs PBS, 65.2%; *P* < .05), with fewer mice developing pancreatic NETs (pasireotide, 86.9% vs PBS, 96.9%; *P* < .05) and smaller increases in pituitary NET volumes (pre-treated vs post-treated, 0.803 ± 0.058 mm^3^ vs 2.872 ± 0.728 mm^3^ [pasireotide] compared with 0.844 ± 0.066 mm^3^ vs 8.847 ±1.948 mm^3^ [PBS]; *P* < .01). In addition, pasireotide-treated mice had fewer pancreatic NETs compared with PBS-treated mice (2.36 ± 0.25 vs 3.72 ± 0.32, respectively; *P* < .001), with decreased proliferation in pancreatic NETs (pasireotide, 0.35 ± 0.03% vs PBS, 0.78 ± 0.08%; *P* < .0001) and pituitary NETs (pasireotide, 0.73 ±0.07% vs PBS, 1.81 ± 0.15%; *P* < .0001), but increased apoptosis in pancreatic NETs (pasireotide, 0.42 ± 0.05% vs PBS, 0.19 ± 0.03%; *P* < .001) and pituitary NETs (pasireotide, 14.75 ± 1.58% vs PBS, 2.35 ± 0.44%; *P* < .001). Thus, pasireotide increased survival and inhibited pancreatic and pituitary NET growth, thereby indicating its potential as an anti-proliferative and pro-apoptotic therapy.

Neuroendocrine tumors (NETs) of the enteropancreatic system and anterior pituitary have an annual incidence of 5 per 100 000 and 8 per 100 000 of the population, respectively (1, 2). These NETs may occur as an isolated endocrinopathy, which may be inherited as occurring in familial isolated pituitary adenomas (3), or they may occur as part of a complex hereditary syndrome, such as multiple endocrine neoplasia (MEN) type 1 (MEN1) (1, 2, 4, 5). Thus, in MEN1 which is an autosomal-dominant disorder, pancreatic and pituitary NETs occur in association with parathyroid and adrenal cortical tumors, together with nonendocrine lesions such as lipomas, collagenomas, and angiofibromas (6, 7). Pancreatic NETs (eg, gastrinomas, insulinomas and nonfunctioning tumors) occur in greater than 40% of patients with MEN1, and pituitary NETs (eg, prolactinomas, somatotrophinomas and corticotrophinomas) occur in greater than 35% of patients after the third decade of life (6, 7). In the absence of treatment, these NETs in patients with MEN1 have been reported to be associated with an earlier mortality (8–10). Indeed, 50–70% of patients with MEN1 will die as a result of a malignant tumor process or sequelae of the disease (8–10), and approximately 30–45% of deaths are a result of malignant pancreatic and foregut NETs (9, 11). Current treatments of pancreatic, foregut, and pituitary NETs are unsatisfactory, with a surgical cure for pancreatic NETs being rarely achieved, as the tumors have metastasized in greater than 50% of patients with MEN1 at presentation, and with pituitary NETs being more aggressive and resistant to treatment in patients with MEN1 (4, 12–14).

Few medical treatments with antiproliferative effects (ie, efficacy in inhibiting tumor growth) are available for advanced pancreatic and pituitary NETs. For example the single-receptor-targeted somatostatin analogs (SAs), octreotide and lanreotide, which act predominantly via the somatostatin receptor type 2 (SSTR2), have been reported to be effective in decreasing pituitary NET GH secretion and growth in up to 70 and 75% of patients with acromegaly, respectively (15–19). In addition, octreotide and lanreotide have been reported to control symptoms and increase progression-free survival (PFS) in 65–80% and approximately 65% of patients with enteropancreatic NETs, respectively (20–23). Pasireotide, which is a multiple-receptor-targeted SA that acts via SSTR1, 2, 3, and 5, has also been shown to have antisecretory and antiproliferative affects on pituitary and enteropancreatic NETs (22, 24–28). Moreover, pasireotide has been reported to be more effective than octreotide in controlling GH hypersecretion in patients with acromegaly (26, 27), and to be also effective in patients with acromegaly who were resistant to treatment with octreotide or lanreotide (28). However, the efficacy and survival benefit of these antitumor effects of SAs in pancreatic and pituitary NETs remains unknown. We therefore assessed the antiproliferative effects of the multireceptor-targeted SA pasireotide (24) in pancreatic and pituitary NETs that develop in an established mouse model for MEN1 (29), in which mice deleted for an allele of the *Men1* gene (ie, heterozygous (*Men1*^+/−^) mice) develop pancreatic and pituitary NETs in association with parathyroid and adrenal tumors. Moreover, *Men1*^+/−^ mice with these pancreatic and pituitary NETs, which develop at 12–15 months of age, have a significantly higher mortality of greater than 30% compared with 10% in wild-type (*Men1*^+/+^) mice (29). Furthermore, the pancreatic and pituitary NETs of *Men1*^+/−^ mice have been reported to express SSTR2 (29), which is similar to SSTR expression in human pancreatic and pituitary NETs. Thus, the pancreatic and pituitary NETs of *Men1*^+/−^ mice are representative of human NETs and the human disorder, MEN1. We therefore used these *Men1*^+/−^ mice in a preclinical trial to determine the effects of pasireotide, which has high affinity for SSTR1, 2, 3, and 5, on the proliferation of pancreatic and pituitary NETs and survival.

## Materials and Methods

### Animals

**Figure 1.**
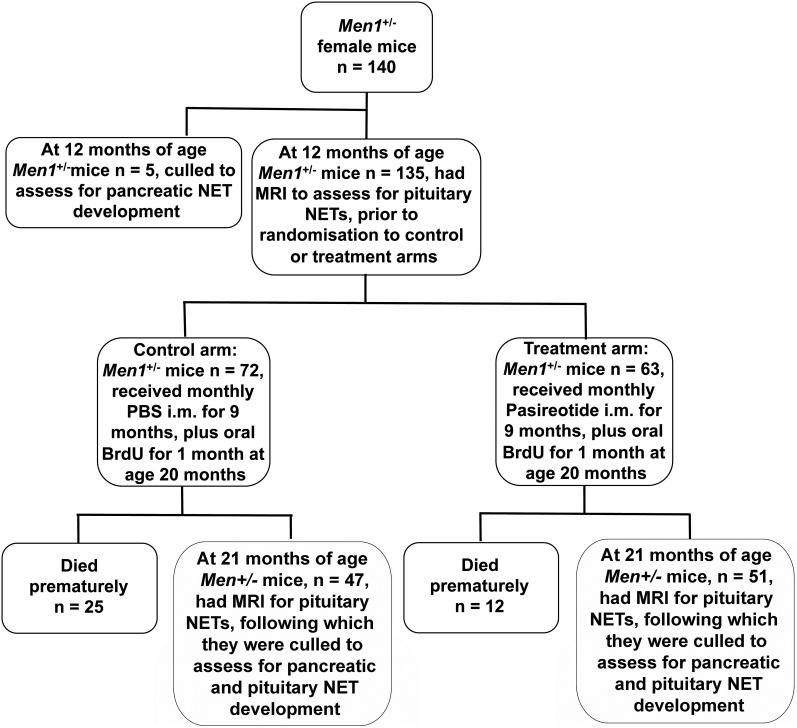
Flowchart for the trial of pasireotide in the treatment of neuroendocrine tumors (NETs) of *Men1*^+/−^ mice. Female *Men1*^+/−^ mice (n = 140) were aged to 12 mo, and 5 *Men1*^+/−^ mice (control nontreated 12-mo group) were culled to assess for the number of pancreatic NETs. The remaining 135 *Men1*^+/−^ mice underwent MRI for assessment of pituitary NETs, and were randomly assigned to a control group given PBS (n = 72) or a treatment group given pasireotide (n = 63) for 9 mo. Mice were also given oral BrdU for 4 wk, commencing at age 20 mo. During the 9 mo from ages 12–21 mo, 25 *Men1*^+/−^ mice from the PBS-treated group and 12 *Men1*^+/−^ mice from the pasireotide-treated group died. Thus, 47 *Men1*^+/−^ mice from the PBS-treated group and 51 *Men1*^+/−^ mice from the pasireotide-treated group completed the study to age 21 mo, when the mice were reassessed using MRI for changes in pituitary NET volume prior to being culled. Pituitary and pancreatic NETs were collected at necropsy.

Animal studies were approved by the University of Oxford Ethical Review Committee and were licensed under the Animal (Scientific Procedures) Act 1986, issued by the United Kingdom Government Home Office Department (PPL30/2914). Female *Men1*^+/−^ mice maintained on a C57BL/6 background were bred and genotyped as previously described (29). Female *Men1*^+/−^ mice were selected for the study as they have a higher occurrence of pituitary NETs when compared with males (pituitary NETs in females ∼45% vs < 5% in males), and also have a similar occurrence of pancreatic NETs (pancreatic NETs in females = 55 vs 45% in males) (29). This strategy enabled a reduction in the numbers of mice used for the study, thereby complying with humane experimental techniques and the principles of reduction, refinement, and replacement (“The Three Rs”) that are recommended for research studies using animals (30). The female *Men1*^+/−^ mice (n = 140) were aged to 12 months, as previously described (29) for the study (Figure 1). At 12 months of age and prior to treatment, five *Men1*^+/−^ mice (control nontreated 12-mo group) were culled and pancreata collected to assess the number of pancreatic NETs. The remaining 135 mice underwent magnetic resonance imaging (MRI) under general anesthesia, to identify pituitary NETs and their volumes (31), and were randomly assigned to a control or treatment arm. The control group (n = 72) received monthly PBS im injections for 9 months (Figure 1), and the treatment group (n = 63) received monthly pasireotide long-acting release (LAR) formulation 40 mg/kg im for 9 months. The pasireotide (SOM230LAR) was stored at 4°C and dissolved in 0.9% sterile saline at 20 mg/mL immediately prior to administration, and the dose of 40 mg/kg was based on previously published pharmacokinetic data in mice (32, 33). These pharmacokinetic studies reported that 80 mg/kg SOM230 LAR, administered parenterally, resulted in average plasma levels of 10–100 ng/mL during the 28-day treatment period, and that parenteral doses of pasireotide LAR up to 320 mg/kg per month were well tolerated in mice. Thus, 40 mg/kg pasireotide LAR given im to mice was expected to be well tolerated and to result in continuously elevated plasma concentrations above the therapeutically effective plasma concentration, which been reported to be 7.8 ng/mL for patients with Cushing's disease treated with pasireotide sc (34) for a month. Monthly weights were recorded from 12 months of age as pasireotide treatment in patients with enteropancreatic NETs and carcinoid syndrome has been reported to be associated with weight loss in greater than 40% of patients. This weight loss is not related to the dose of pasireotide or tumor progression (35). Mortality was also recorded from 12 months of age. Mice were fed standard diet (Rat and Mouse No. 1, expanded diet, Special Diet Services, Ltd) and provided with water ad libitum, which from 20 months of age contained 5-bromo-2-deoxyuridine (BrdU), 1 mg/mL, kept in foil-wrapped bottles, to label proliferating cells, as previously described (36). The BrdU water was changed twice weekly for 4 weeks. Four weeks later at 21 months of age, *Men1*^+/−^ mice underwent a further MRI scan to assess for changes in pituitary NET volumes, after which necropsy was performed to also assess for the burden of pancreatic and pituitary NETs and to collect NETs.

### MRI

Mice were anesthetized with isoflurane and MRI of the cranium undertaken using a nonionic MRI contrast medium, gadodiamide (Omniscan, Amersham Health AS), which was injected intraperitoneally at 0.1 mmol/kg. An 11.7 Tesla (500 MHz) MR system was used, image reconstruction was performed using purpose-written software in Matlab (MathWorks), and image data exported into tagged image file format (TIFF) and loaded into Scion Image (Scion Corporation). Pituitary tumor volumes using MRIcro software were quantified as the sum of the area of all 1-mm sections containing the tumor (31).

### Histology and immunohistochemistry

Pancreatic and pituitary tissues were dissected from mice and perfusion fixed with 4% paraformaldehyde and embedded in paraffin (31, 36). Sections (5 μm) were dewaxed, hydrated, and stained with hematoxylin and eosin (H&E) (31, 36). For immunohistochemistry, sections underwent antigen retrieval at 121°C in citrate solution (Antigen Unmasking Solution; Vector Laboratories) followed by blocking with 10% donkey serum/PBS for 30 minutes before primary antibody incubation. Primary antibodies were rat anti-BrdU (Abcam, Cambridge), guinea pig anti-insulin (Abcam), rabbit antiprolactin (National Hormone and Peptide Program [NHPP]), rabbit antigrowth hormone (NHPP), rabbit anti-SSTR1 (Biorbyt), rabbit anti-SSTR2 (Abcam), rabbit anti-SSTR3 (Antibodies Online), and rabbit anti-SSTR5 (Antibodies Online) (Table 1) (29, 31, 36).

**Table 1.** Antibody Table

Peptide/Protein Target	Antigen Sequence (if known)	Name of Antibody	Manufacturer, Catalog Number, and/or Name of the Institution Providing the Antibody	Species Raised in; Monoclonal or Polyclonal	Dilution Used
BrdU		BU1/75 (ICR1)	Abcam (Cambridge, UK), ab6326	Rat monoclonal	1:100
Insulin		ab7842	Abcam (Cambridge, UK), ab7842	Guinea pig polyclonal	1:100
Prolactin			National Hormone and Peptide Program (Torrence CA)	Rabbit polyclonal	1:200
GH			National Hormone and Peptide Program (Torrence CA)	Rabbit polyclonal	1:100
Somatostatin receptor 1		orb11421	Biorbyt (Cambridge, UK), orb11421	Rabbit polyclonal	1:100
Somatostatin receptor 2	C-ERSDSKQDKSRLNETTETQRT	ab9550	Abcam (Cambridge, UK), ab9550	Rabbit polyclonal	1:100
Somatostatin receptor 3		ABIN685552	Antibodies Online (Aachen, Germany), ABIN685552	Rabbit polyclonal	1:100
Somatostatin receptor 5		ABIN738231	Antibodies Online (Aachen, Germany), ABIN738231	Rabbit polyclonal	1:100
guinea pig IgG (H+L)		Cy2 AffiniPure Donkey Anti-Guinea Pig IgG (H + L)	Jackson ImmunoResearch (West Grove, PA), 706-225-148	Donkey polyclonal	1:100
Rabbit IgG (H+L)		Cy2 AffiniPure Donkey Anti-Rabbit IgG (H + L)	Jackson ImmunoResearch (West Grove, PA), 711-225-152	Donkey polyclonal	1:100
Rat IgG (H+L)		Cy3 AffiniPure Donkey Anti-Rat IgG (H + L)	Jackson ImmunoResearch (West Grove, PA), 712-165-153	Donkey polyclonal	1:250
Rabbit IgG (H+L)		Peroxidase AffiniPure Donkey Anti-Rabbit IgG (H + L)	Jackson ImmunoResearch (West Grove, PA), 711-035-152	Donkey polyclonal	1:500

Abbreviation: H+L, Heavy and light chain.

Secondary antibodies were Cy2-conjugated donkey antiguinea pig (Jackson ImmunoResearch) and donkey antirabbit (Jackson ImmunoResearch), Cy3-conjugated donkey antirat (Jackson ImmunoResearch), or horseradish peroxidase–conjugated donkey antirabbit (Jackson ImmunoResearch) with a peroxidase/3,3′-diaminobenzidine detection system (Vector Laboratories). Counterstaining was performed with Hematoxylin QS (Vector Laboratories); alternatively, nuclear counterstaining was performed with 4′,6-diamino-2-phenylindole (DAPI) (ProLong Gold Antifade reagent with DAPI; Molecular Probes). Sections were viewed by light or fluorescent microscopy using an Eclipse E400 microscope (Nikon), and images captured at 100–400× resolution using a DXM1200C digital camera and NIS-Elements BR 2.30 software (both Nikon). Proliferation rates of pancreatic NET cells were calculated as mean ± SEM, from more than six pancreatic NETs, from each of more than or equal to six animals per treatment group, and those for pituitary NETs were calculated as mean ± SEM, from each of more than or equal to six animals per treatment group. Daily proliferation rates, expressed as a percentage, were calculated by dividing total rates by the number of days of continuous BrdU administration, as previously described (31, 36). Apoptosis was assessed using the terminal deoxyuridine, 5′-triphosphate nick end labeling (TUNEL) assay (ApopTag Fluorescein In Situ Apoptosis Detection kit, Chemicon). Apoptotic rates of pancreatic NET cells were calculated as mean ± SEM, from greater than or equal to four pancreatic NETs, from each of greater than or equal to three animals per treatment group, and those for pituitary NETs were calculated as mean ± SEM, from each of greater than or equal to four fields of view from five animals per treatment group, and expressed as a percentage of labeled cells (31, 36).

### Statistics

Parametric data were compared for statistical significance using the Student *t* test, categorical data analyzed by Fisher's exact test, and survival compared using the log-rank Mantel-Cox test and Kaplan-Meier analysis, as described previously (29, 31, 36).

## Results

### Expression of somatostatin receptors in pancreatic and pituitary NETs

**Figure 2.**
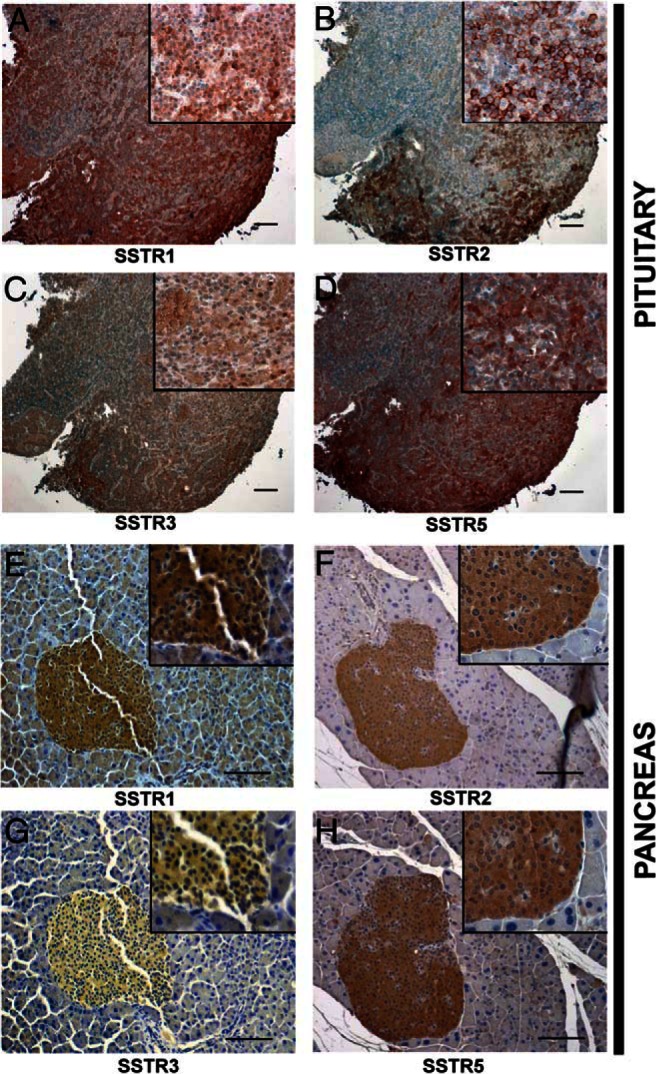
Immunohistochemical analysis of SSTR expression in pituitary and pancreatic NETs. A–D, pituitary NETs; and E-H, pancreatic NETs. A, Pituitary NET showing diffuse cytoplasmic SSTR1 staining in a large number of cells. B, Pituitary NET showing moderate SSTR2 staining of the plasma membrane in a subset of cells. C, Pituitary NET showing diffuse cytoplasmic SSTR3 staining in a minority of cells. D, Pituitary NET showing moderate cytoplasmic SSTR5 staining in a large number of cells. E, Pancreatic NET showing cytoplasmic SSTR1 staining. F, Pancreatic NET showing cytoplasmic SSTR2 staining. G, Pancreatic NET showing absence of SSTR3 staining. H, Pancreatic NET showing cytoplasmic SSTR5 staining in most islet cells. Thus, pituitary NETs show expression of SSTR1, 2, 3, and 5, whereas pancreatic NETs show expression of only SSTR1, 2, and 5. Magnification 100× for pituitary NETs and 200x for pancreatic NETs; inset magnification 400× for pituitary and pancreatic NETs. Scale bar = 100 μm.

Pasireotide, a multireceptor-targeted SA, has high affinity for SSTR1, 2, 3, and 5. Expression of SSTRs in pancreatic and pituitary NETs that developed in the *Men1*^+/−^ mice were therefore investigated using immunohistochemical analysis. Analysis of the pituitary NETs from 19 *Men1*^+/−^ mice revealed expression of at least one SSTR subtype in 95% of tumors. The expression level of each SSTR subtype varied between pituitary NETs with SSTR3 generally lower than SSTR1, 2, and 5 (Figure 2, A–D). Overall, SSTR1 expression was observed in approximately 95% of tumors, SSTR2 in greater than 45% of tumors, SSTR3 in greater than 40% of tumors, and SSTR5 in approximately 75% of tumors. Analysis of pancreatic NETs from 10 *Men1*^+/−^ mice revealed them to express SSTR1, 2, and 5 but not SSTR3 (Figure 2, E–H).

### Health and survival of pasireotide-treated *Men1*^+/−^ mice

**Figure 3.**
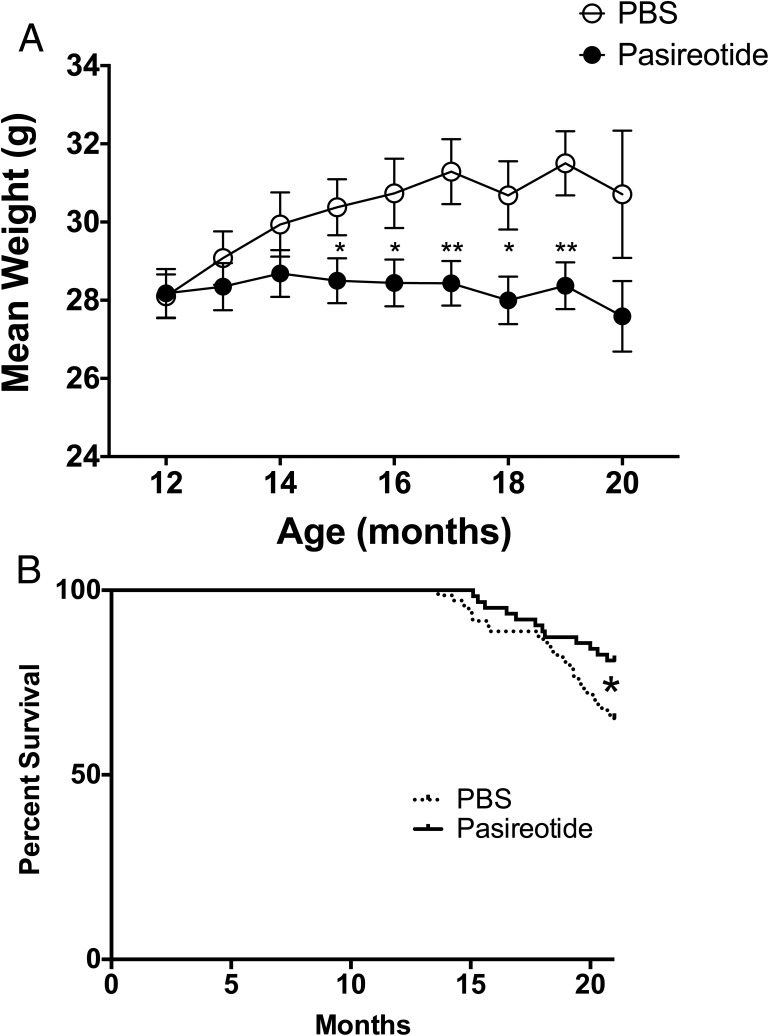
Weight and survival in *Men1*^+/−^ mice. A, Mean monthly weight of mice in the PBS-treated (open circles) and pasireotide-treated (closed circles) groups (n = 24–70). The pasireotide-treated *Men1*^+/−^ mice did not gain weight when compared with the PBS-treated *Men1*^+/−^ mice (*, *P* < .05; **, *P* < .01). Error bars represent SEM. B, Kaplan-Meier analysis of mortality rates in *Men1*^+/−^ mice during the 21 mo of study. Forty-seven of 72 PBS-treated *Men1*^+/−^ mice (65.2%) survived until age 21 mo, compared with 51 of 63 pasireotide-treated mice (80.9%). Thus, survival in pasireotide-treated *Men1*^+/−^ mice was significantly higher at age 21 mo, compared with control PBS-treated *Men1*^+/−^ mice (*P* < .05, log-rank Mantel-Cox test).

The PBS-treated *Men1*^+/−^ mice continued to gain body weight between the ages of 12 and 17 months, whereas the pasireotide-treated *Men1*^+/−^ mice maintained a stable body weight. Thus, at age 15 months the PBS-treated mice had a significantly higher mean body weight (±SEM) of 30.4 ± 0.7 g compared with 28.5 ± 0.6 g for pasireotide-treated mice (*P* < .05), and at 19 months of age this difference was greater (mean ± SEM body-weight, 31.5 ± 0.7 g for PBS-treated mice vs 28.4 ± 0.5 g for pasireotide-treated mice; *P* < .01) (Figure 3A). Despite the absence of weight gain in the pasireotide-treated group, the overall health of the pasireotide-treated *Men1*^+/−^ mice, which was assessed by body condition scores, grimace, and coat condition (37–39), was similar to that of the PBS-treated *Men1*^+/−^ mice, with the exception of the coat condition (assessed by piloerection), which was significantly better in the pasireotide-treated mice than the PBS-treated *Men1*^+/−^ mice (30.6% for PBS-treated mice vs 10.6% for pasireotide-treated mice; *P* < .05) (Supplemental Table 1 and Supplemental Figure 1). Moreover, the pasireotide-treated *Men1*^+/−^ mice had a significantly increased survival of 80.9% when compared with that of PBS-treated *Men1*^+/−^ mice, which was 65.2% at 21 months of age (*P* < .05, Figure 1 and 3B). Indeed, the >80% survival of the pasireotide-treated *Men1*^+/−^ mice at age 21 months is comparable to the approximate 85% survival reported in 21-month-old wild-type *Men1*^+/+^ mice (29).

### Pasireotide decreases occurrence of pancreatic NETs

Pancreatic NET occurrence assessed at necropsy was significantly lower in *Men1*^+/−^ mice treated with pasireotide when compared with *Men1*^+/−^ mice treated with PBS (86.9% of pasireotide-treated mice [53 of 61] vs 96.9% of PBS-treated mice [63 of 65]; *P* < .05). Furthermore, the mean number of NETs per pancreas in the 21-month-old pasireotide-treated *Men1*^+/−^ mice was 2.36 ± 0.25 (n = 51) and similar to 2.60 ± 1.17 (n = 5) observed in the 12-month-old *Men1*^+/−^ mice, but significantly less than the 3.72 ± 0.32 (n = 47) (*P* < .001) observed in 21-month-old PBS-treated *Men1*^+/−^ mice. Thus, the pasireotide-treated *Men1*^+/−^ mice had fewer NETs per pancreas and fewer pancreatic NETs per mouse than the PBS-treated *Men1*^+/−^ mice, which was in part due to the absence of development of new NETs. There was no difference in the occurrence of pituitary NETs between the pasireotide-treated and PBS-treated groups, when analyzing those surviving to 21 months of age (64.7% pasireotide-treated *Men1*^+/−^ mice vs 68.1% PBS-treated *Men1*^+/−^ mice) or when those that died prematurely were included (64.5% pasireotide-treated *Men1*^+/−^ mice vs 67.2% PBS-treated *Men1*^+/−^ mice). However, there was a difference in pituitary NET size between the pasireotide-treated and control PBS-treated groups, as detailed below.

### Pasireotide decreases size of pituitary NETs

**Figure 4.**
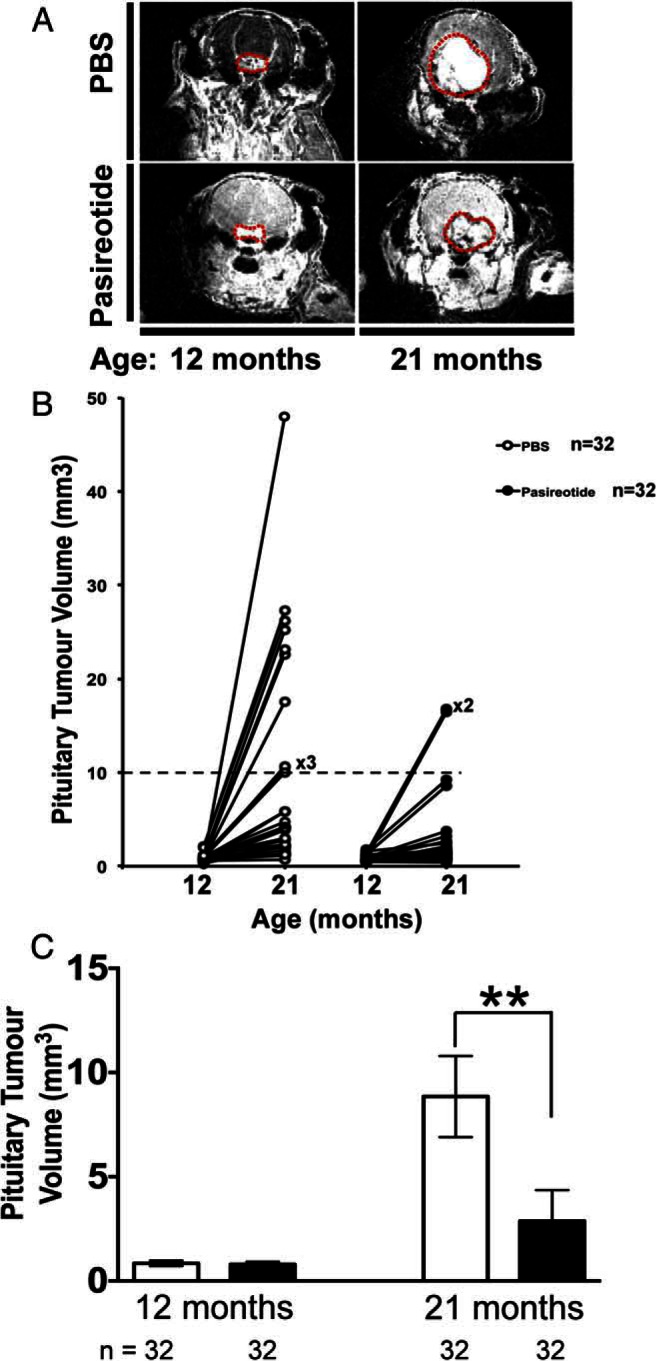
Pituitary NET imaging and volumetric measurements. A, Cranial MRI with gadolinium enhancement demonstrating pituitary NETs (outlined in red dotted line) in a PBS-treated (upper panels) *Men1*^+/−^ mouse and a pasireotide-treated (lower panels) *Men1*^+/−^ mouse at age 12 and 21 mo. B, MRI was used to assess pituitary NET volumes, pretreatment (12 mo) and 9 mo post-treatment (21 mo). The PBS-treated *Men1*^+/−^ mice (n = 32) and pasireotide-treated *Men1*^+/−^ mice (n = 32) had similarly sized pituitary NETs of approximately 0.3–3.0 mm^3^ at age 12 mo (baseline). However by age 21 mo, 10 of 32 of PBS-treated *Men1*^+/−^ mice (31%) had developed large pituitary NETs of at least 10 mm^3^, whereas only two of 32 of *Men1*^+/−^ mice (6.3%) treated with pasireotide had developed pituitary NETs with volumes at least 10 mm^3^. There was no evidence of pituitary NET development in the remaining 15 of 47 PBS-treated *Men1*^+/−^ mice (31.9%) or 19 of pasireotide-treated *Men1*^+/−^ mice 51 (37.3%) at age 21 mo, consistent with our previous observation that pituitary NETs only develop in a subset of female *Men1*^+/−^ mice (29). C, The mean pituitary NET volumes at 12 mo in the PBS-treated (open bars) (n = 32) and pasireotide-treated (filled bars) (n = 32) *Men1*^+/−^ mice were not significantly different (mean volume ± SEM of PBS-treated, 0.844 ± 0.066 mm^3^ vs pasireotide-treated, 0.803 ± 0.058 mm^3^). However, after 9 mo of treatment, at the age of 21 mo, the pasireotide-treated mice (n = 32) had a significantly smaller increase in pituitary NET volume when compared with the PBS-treated *Men1*^+/−^ mice, such that the mean ± SEM pituitary tumor volume of the pasireotide-treated *Men1*^+/−^ mice was 2.872 ± 0.728 mm^3^ compared with 8.847 ± 1.948 mm^3^ of the PBS-treated *Men1*^+/−^ mice (**, *P* < .01). Error bars represent SEM.

Pituitary NET volume, assessed by MRI at 12 months and 21 months of age (Figure 4A), was used as a measure of tumor growth and progression. There was no difference in pituitary NET volume (measured in cubic millimetres) between PBS-treated and pasireotide-treated *Men1*^+/−^ mice at 12 months of age. However, at 21 months of age the PBS-treated *Men1*^+/−^ mice had significantly larger pituitary NETs than the pasireotide-treated mice (*P* < .01) (Figure 4, B and C). Thus, at 21 months of age 31.3% of PBS-treated *Men1*^+/−^ mice (10/32) had pituitary NETs at least 10 mm^3^, whereas only 6.3% of pasireotide-treated *Men1*^+/−^ mice (2/32) had pituitary NETs at least 10 mm^3^ (*P* < .01, Fisher's exact test). The mean (±SEM) pituitary NET volume in PBS-treated *Men1*^+/−^ mice increased between 12 and 21 months of age from 0.844 ± 0.066 to 8.847 ± 1.948 mm^3^ whereas in the pasireotide-treated *Men1*^+/−^ mice, the increase was smaller, from 0.803 ± 0.058 to 2.872 ± 0.728 mm^3^. These results suggest that pasireotide treatment significantly reduces pituitary NET growth in *Men1*^+/−^ mice.

### Pasireotide decreases cell proliferation and increases apoptosis in pancreatic and pituitary NETs

**Figure 5.**
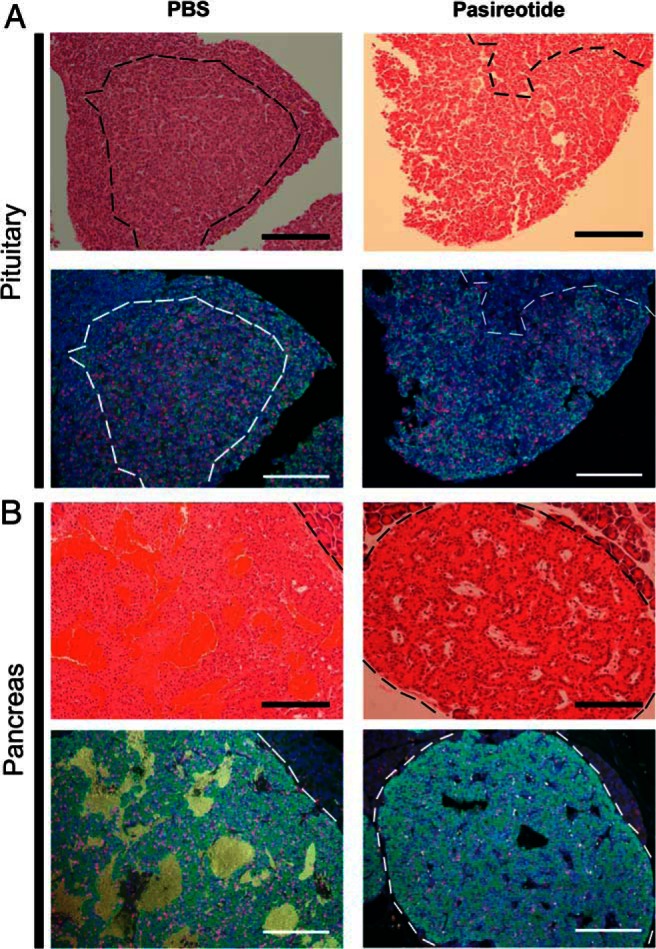
Assessment by immunohistochemical analysis of effects of pasireotide on proliferation in *Men1*^+/−^ mice pituitary NETs and pancreatic NETs. Pituitary and pancreatic NETs obtained from control PBS-treated *Men1*^+/−^ mice are compared with pasireotide-treated *Men1*^+/−^ mice, after administration of BrdU for 4 wk. A, Pituitary NET-adjacent sections from *Men1*^+/−^ mice stained with H&E and immunostained for prolactin (green) and BrdU (red). Nuclei were counterstained with DAPI (Blue). The NETs are circled by dashed lines (black in the H&E sections and white in the fluorescent immunohistochemical sections). The large pituitary NETs from a PBS-treated *Men1*^+/−^ mouse and a pasireotide-treated *Men1*^+/−^ mouse showing immunostaining for prolactin but not GH (data not shown), are highly proliferating (red nuclei), although there are fewer proliferating cells in the pituitary NETs of the pasireotide-treated mouse. B, Pancreatic NET-adjacent sections from *Men1*^+/−^ mice stained with H&E and immunostained for insulin (green) and BrdU (red), and counterstained for nuclei (blue). The NETs are circled by dashed lines (black in the H&E sections and white in the fluorescent immunohistochemical sections). The pancreatic NET from the pasireotide-treated *Men1*^+/−^ mouse had fewer proliferating cells. Scale bar = 100 μm.

**Figure 6.**
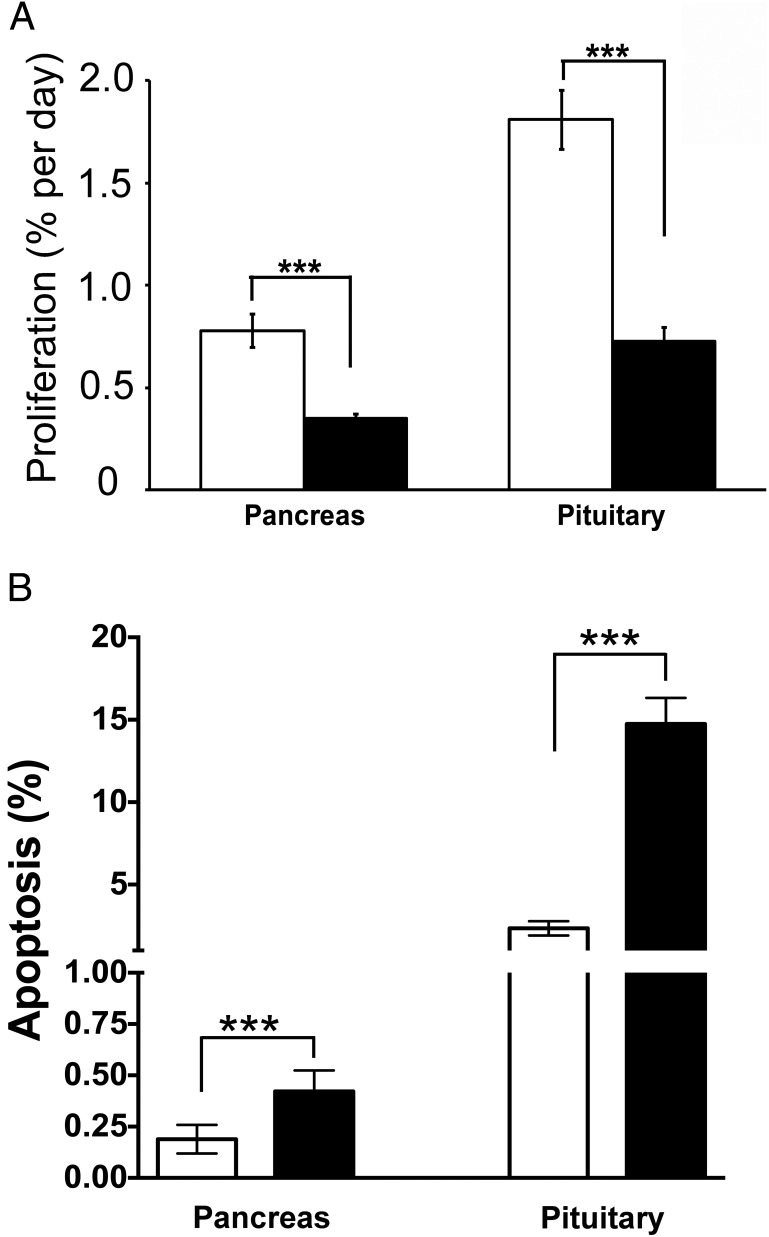
Proliferation and apoptosis of pancreatic and pituitary NETs in *Men1*^+/−^ mice. Proliferative and apoptotic rates were assessed by BrdU labeling and TUNEL assays, respectively. Pancreatic and pituitary NETs were collected from *Men1*^+/−^ mice age 21 mo given BrdU for 28 d before necropsy. The proliferation and apoptotic rates were compared between PBS and pasireotide-treated mice. A, Proliferation rates in pancreatic and pituitary NETs from PBS-treated *Men1*^+/−^ mice (open bars) (n = 36 sections from six mice) and pasireotide-treated *Men1*^+/−^ mice (filled bars) (n = 36 sections from six mice). B, Apoptotic rates in pancreatic and pituitary NETs from PBS-treated *Men1*^+/−^ mice (open bars) (n = 19 pancreatic NET sections from three mice, and 40 pituitary NET sections from five mice) and pasireotide-treated *Men1*^+/−^ mice (filled bars) (n = 21 pancreatic NET sections from five mice, and 39 pituitary NET sections from six mice). Pancreatic and pituitary NETs demonstrated a significant 4-fold increase in apoptosis with pasireotide treatment when compared with PBS-treatment. Error bars represent SEM; ***, *P* < .001.

Pituitary NETs were predominantly prolactinomas (Figure 5A) but somatotrophinomas and nonfunctioning adenomas were also observed, whereas pancreatic NETs predominantly immunostained for insulin (Figure 5B). Sections of pancreatic and pituitary NETs from PBS-treated and pasireotide-treated *Men1*^+/−^ mice, which had received 4 weeks of oral BrdU, were used for immunohistochemical analysis of BrdU incorporation into dividing cells and daily proliferation rates determined (Figure 5). Fewer proliferating cells were observed in the pituitary NETs (Figure 5A) and pancreatic NETs (Figure 5B) of pasireotide-treated *Men1*^+/−^ mice, and this was confirmed by calculation of the mean proliferation rates. Thus, in pancreatic NETs the mean (±SEM) proliferation rate in pasireotide-treated *Men1*^+/−^ mice was significantly lower than that in PBS-treated *Men1*^+/−^ mice (0.35 ± 0.03 vs 0.78 ± 0.08% per day; *P* < .0001; Figure 6A). Similarly, in pituitary NETs the mean (±SEM) proliferation rate in pasireotide-treated *Men1*^+/−^ mice was significantly lower than that in PBS-treated *Men1*^+/−^ mice (0.73 ± 0.07 vs 1.81 ± 0.15% per day; *P* < .0001; Figure 6A). Thus, daily NET proliferation rates were more than 2-fold lower in pasireotide-treated mice. The rate of apoptosis in the pancreatic and pituitary NETs was assessed by TUNEL analysis, and in pancreatic NETs the mean (±SEM) apoptosis rate in pasireotide-treated *Men1*^+/−^ mice was significantly higher than that in PBS-treated *Men1*^+/−^ mice (0.42 ± 0.05 vs 0.19 ± 0.03%; *P* < .001; Figure 6B). Similarly, in pituitary NETs the mean (±SEM) apoptosis rate in pasireotide-treated *Men1*^+/−^ mice was significantly higher than that in PBS-treated *Men1*^+/−^ mice (14.75 ± 1.58 vs 2.35 ± 0.44%; *P* < .001; Figure 6B). Thus, the apoptotic rate was more than 2-fold higher in pancreatic NETs and more than 6-fold higher in pituitary NETs of pasireotide-treated *Men1*^+/−^ mice.

## Discussion

Our results show that pasireotide, a multireceptor-targeted SA with high affinity for SSTR1, 2, 3, and 5 is effective in reducing proliferation and increasing apoptosis of pancreatic and pituitary NETs (Figures 5 and 6) that develop in a mouse model for MEN1. These findings, which agree with the reported antisecretory, antiproliferative, and proapoptotic actions of pasireotide on pancreatic NETs that developed in a MEN1 conditional knockout mouse model (40), suggest that pasireotide may represent an important option for antitumor therapy of NETs in patients with MEN1. In addition, our results suggest that pasireotide may inhibit the development of pancreatic NETs in *Men1*^+/−^ mice, thereby suggesting that it may also have a chemopreventative role in the treatment of MEN1-associated NETs. This potentially expands the range of indications for the use of pasireotide as an antitumor drug. For example, pasireotide, which has been reported to be effective in treating GH/prolactin-secreting adenomas that developed in transgenic mice ubiquitously overexpressing the high mobility group family AT-hook2 (*HMGA2*) gene (25), has been used in Phase III clinical trials to treat acromegaly, in which pasireotide was effective in suppressing GH secretion to less than 2.5 μg/L with normalization of IGF-I levels and significant tumor reduction in approximately 75% of patients (27, 41). In other Phase III clinical trials involving patients with Cushing's disease, pasireotide treatment significantly reduced cortisol excess, with improvement in clinical symptoms and signs (42, 43). In a Phase II clinical trial of patients with NETs and carcinoid syndrome, pasireotide controlled flushing and diarrhea in approximately 25% of patients (35); and in another single arm Phase II clinical trial of pasireotide in patients with a heterologous population of metastatic pancreatic and extrapancreatic NETs, pasireotide was reported to produce a median PFS of 11 months (24), which compares favorably with a PFS of approximately 5 months observed in the placebo arms of other pancreatic NET trials (21, 44).

These antitumor effects of pasireotide have similarities to those reported for octreotide and lanreotide (20, 21). Thus, octreotide and lanreotide, which suppress hormone secretion, have also been reported to exert antiproliferative effects in patients with somatotrophinomas causing acromegaly (15–19), midgut NETs (21), and enteropancreatic NETs (20). Octreotide and lanreotide act predominantly, if not exclusively on SSTR2, whereas pasireotide acts on SSTR1, 2, 3, and 5, and has a slightly lower activity at SSTR2, but considerably greater activity at SSTR1, 3, and especially 5 (45), and this wider range of pasireotide activity on SSTRs likely facilitates its efficacy on NETs that are refractory or resistant to other SAs, such as octreotide and lanreotide (35). Moreover, the efficacy of pasireotide in treating aggressive NETs, which have lost responsiveness to octreotide or lanreotide, may also be due to a reduction in SSTR2 expression in such aggressive NETs when compared with less-aggressive NETs (46). Pasireotide has been reported to have in vitro effects that are similar to those of octreotide on cell survival, chromogranin A, and SSTR2 phosphorylation and trafficking in human pancreatic NET cells (47), but to have greater in vivo inhibitory effects compared to octreotide, on pituitary tumor growth in the HMGA2 transgenic mouse model (25); and in controlling symptoms of carcinoid syndrome in more than 25% of patients with advanced NETs that were refractory or resistant to octreotide LAR therapy (35). The antitumor effects of these SAs have been reported to involve direct actions on the cell cycle and apoptosis as well as indirect inhibitory actions on angiogenesis and tumor growth factors (48).

The antiproliferative and proapoptotic actions of pasireotide on the pancreatic and pituitary NETs (Figures 5 and 6) of *Men1*^+/−^ mice, which included reduced pituitary NET size (Figure 4), were associated with higher survival (Figure 3B) in the pasireotide-treated *Men1*^+/−^ mice. However, the pasireotide-treated *Men1*^+/−^ mice did not gain in body weight, unlike the PBS-treated *Men1*^+/−^ mice, which gained weight (Figure 3A), as reported to occur in female C57BL/6 mice until 15–20 months of age (49, 50). These inhibitory effects of pasireotide on body weight gain in the *Men1*^+/−^-treated mice, are consistent with previous reports in rodents (22) and in NET patients, of whom more than 40% lost 10–20% of body weight (35). These effects on body weight of pasireotide may be partly explained by its inhibitory effects on GH and IGF-I (22), and also on its ability to induce hyperglycemia in rodents and humans (22, 32, 35). Thus, recent studies in male Siberian hamsters (*Phodopus sungorus*) under a long-day photoperiod, which investigated the mechanisms of pasireotide-induced weight loss, have shown that pasireotide treatment resulted in a reduction of circulating IGF-I, indicative of a suppression of GH secretion, and that this was accompanied by increased GH releasing hormone (*Ghrh*) mRNA expression in the arctuate nucleus, and reduced somatostatin mRNA expression in the paraventricular nucleus, consistent with reduced GH feedback to the hypothalamus (51). In addition to these effects of pasireotide on GH and IGF-I, pasireotide has also been reported to be associated with hyperglycemia and weight loss in patients with Cushing's disease (42) or metastatic gastrointestinal NETs (35) that received pasireotide treatment. It has been suggested that the hyperglycemia may be due to the different inhibitory effects of pasireotide on pancreatic α- and β-cells, which produce glucagon and insulin, respectively (52–54). Pancreatic α- and β-cells express high levels of SSTR1, 2, and 5, and the high affinity of pasireotide for SSTR5 will inhibit insulin secretion (52) without affecting glucagon secretion, which is mainly mediated by SSTR2 (32, 53, 54). Furthermore, pasireotide treatment in human healthy volunteers resulted in hyperglycemia that was associated with marked decreases in insulin secretion and incretin responses, but only mild suppression of glucagon secretion and with no effect on insulin sensitivity (55), and pasireotide treatment in mice has also been reported to result in hyperglycemia due to decreased circulating insulin concentrations (40). Thus, it seems possible that pasireotide-induced abnormalities of glucose homeostasis, and of GH and IGF-I may be responsible for the lack of weight gain in the pasireotide-treated *Men1*^+/−^ mice. However, it is important to note that the observed weight loss in patients with metastatic gastrointestinal NETs who were treated with pasireotide stabilized after 28 weeks and that there was no correlation between the dose of pasireotide and weight loss (35).

In summary, pasireotide treatment in *Men1*^+/−^ mice with pancreatic and pituitary NETs was associated with reduced proliferation and increased apoptosis of NETs which resulted in approximately 20% improvement in survival. Pasireotide also likely resulted in chemoprevention of NET development. Thus, these studies suggest the potential utility of SAs such as pasireotide for the treatment of pancreatic and pituitary NETs in patients with MEN1.
